# Diagnostic performance of photon-counting detector CT for differentiation between adrenal adenomas and metastases

**DOI:** 10.1007/s00330-024-10675-x

**Published:** 2024-03-14

**Authors:** Stefanie Bette, Franka Risch, Luca Canalini, Judith Becker, Eva V. Leithner, Adrian Huber, Mark Haerting, Bertram Jehs, Claudia Wollny, Florian Schwarz, Kartikay Tehlan, Christian Scheurig-Muenkler, Thomas Wendler, Thomas Kroencke, Josua A. Decker

**Affiliations:** 1 Diagnostic and Interventional Radiology, University Hospital Augsburg, Faculty of Medicine, University of Augsburg, Stenglinstr. 2, 86156 Augsburg, Germany; 2 Diagnostic and Interventional Radiology, Donau-Isar-Klinikum, Perlasberger Str. 41, 94469 Deggendorf, Germany; 3 Institute of Digital Health, University Hospital Augsburg, Augsburg, Germany; 4 Computer-Aided Medical Procedures and Augmented Reality, School of Computation, Information and Technology, Technical University of Munich, Munich, Germany; 5 Centre for Advanced Analytics and Predictive Sciences (CAAPS), University of Augsburg, Universitätsstr. 2, 86159 Augsburg, Germany

**Keywords:** Photon-counting detector computed tomography, Adrenal adenomas, Virtual non-contrast

## Abstract

**Objectives:**

Aim of this study was to assess the value of virtual non-contrast (VNC) reconstructions in differentiating between adrenal adenomas and metastases on a photon-counting detector CT (PCD-CT).

**Material and methods:**

Patients with adrenal masses and contrast-enhanced CT scans in portal venous phase were included. Image reconstructions were performed, including conventional VNC (VNC_Conv_) and PureCalcium VNC (VNC_PC_), as well as virtual monochromatic images (VMI, 40–90 keV) and iodine maps. We analyzed images using semi-automatic segmentation of adrenal lesions and extracted quantitative data. Logistic regression models, non-parametric tests, Bland–Altman plots, and a random forest classifier were used for statistical analyses.

**Results:**

The final study cohort consisted of 90 patients (36 female, mean age 67.8 years [range 39–87]) with adrenal lesions (45 adenomas, 45 metastases). Compared to metastases, adrenal adenomas showed significantly lower CT-values in VNC_Conv_ and VNC_PC_ (*p* = 0.007). Mean difference between VNC and true non-contrast (TNC) was 17.67 for VNC_Conv_ and 14.85 for VNC_PC_. Random forest classifier and logistic regression models both identified VNC_Conv_ and VNC_PC_ as the best discriminators. When using 26 HU as the threshold in VNC_Conv_ reconstructions, adenomas could be discriminated from metastases with a sensitivity of 86.7% and a specificity of 75.6%.

**Conclusion:**

VNC algorithms overestimate CT values compared to TNC in the assessment of adrenal lesions. However, they allow a reliable discrimination between adrenal adenomas and metastases and could be used in clinical routine in near future with an increased threshold (e.g., 26 HU). Further (multi-center) studies with larger patient cohorts and standardized protocols are required.

**Clinical relevance statement:**

VNC reconstructions overestimate CT values compared to TNC. Using a different threshold (e.g., 26 HU compared to the established 10 HU), VNC has a high diagnostic accuracy for the discrimination between adrenal adenomas and metastases.

**Key Points:**

*• Virtual non-contrast reconstructions may be promising tools to differentiate adrenal lesions and might save further diagnostic tests.*

*• The conventional and a new calcium-preserving virtual non-contrast algorithm tend to systematically overestimate CT-values compared to true non-contrast images.*

*• Therefore, increasing the established threshold for true non-contrast images (e.g., 10HU) may help to differentiate between adrenal adenomas and metastases on contrast-enhanced CT.*

## Introduction

The incidental detection of adrenal lesions has increased over the past decades due to improved imaging techniques and increasing numbers of CT scans, with an estimated prevalence of 4–8% on abdominal CT scans [1–4]. Recent guidelines from the American College of Radiology provide an algorithm for the workup of incidentally detected adrenal lesions ≥ 1 cm [4]. Depending on size, the presence of indeterminate or high-risk imaging features, and history of cancer, either a follow-up or an adrenal CT is recommended [4]. An adrenal CT consists of a pre-contrast scan, a portal venous phase, and a late phase (15 min) [4, 5].

Adrenal adenomas can be differentiated from metastases by low CT values on unenhanced CT (< 10 HU) or by rapid washout between portal venous and late contrast phases (relative washout > 60%) [1, 4, 6]. In clinical routine, adrenal lesions are often detected on contrast-enhanced CT scans and therefore cannot be confidently identified as adenomas. In these cases, further imaging (e.g., an adrenal CT) or follow-up is recommended [4], resulting in additional radiation dose to the patient or a delayed clarification with the potential psychological burden respectively.

Previous studies on dual-energy CT (DECT) have addressed the value of virtual non-contrast (VNC) images for the assessment of adrenal lesions [1, 7–9]. However, most studies indicated that VNC overestimated CT values compared to true non-contrast (TNC). VNC series were therefore not recommended for routine clinical use.

In 2021, the photon-counting detector (PCD) technology was introduced. It enables the direct conversion of single X-ray photons into an electric signal, resulting in the reduction of electronic noise and the availability of broad spectral imaging with each scan [10]. A recent study assessed the value of VNC reconstructions on a PCD-CT for the evaluation of adrenal adenomas. However, similar to previous studies, VNC showed an over- or underestimation of CT values compared to TNC [11].

Besides the conventional VNC (VNC_Conv_), a new algorithm is available for creating VNC series: the PureCalcium algorithm (VNC_PC_). Because iodine has a similar attenuation as calcium, calcium contrast is partially removed in VNC_Conv_ series. To address this problem and preserve calcium contrast, VNC_PC_ creates a calcium mask before material differentiation. Recent studies analyzing this algorithm reported more consistent VNC values not only for calcified lesions but also for soft tissue [12–14].

Aims of this study were (i) to analyze the conventional VNC (VNC_Conv_) and the new VNC algorithm (VNC_PC_) for assessment of adrenal lesions in comparison to TNC and (ii) to analyze the value of spectral imaging for the discrimination of adrenal lesions on contrast-enhanced CT scans.

## Material and methods

### Study population

We searched the local database consisting of patients with suspected malignancy and a contrast-enhanced CT of the abdomen between 04/2021 and 09/2022.

The local Medical Research and Ethics Committee (MREC) approved this retrospective single-center study and waived the need for informed consent for the patients included in the retrospective cohort (protocol number: 23–0451).

Inclusion criteria for the retrospective cohort were as follows: (1) age ≥ 18 years, (2) contrast-enhanced CT of the abdomen on a dual-source photon-counting detector CT (PCD-CT) between 04/2021 and 09/2022 in portal venous phase, (3) presence of an adrenal lesion ≥ 1 cm.

### Reference standard

The definition of lesions as adenomas or metastases was performed as described previously [1, 4]. Lesions were diagnosed as adenomas when (1) unenhanced CT showed CT values ≤ 10 HU or (2) size did not change during at least 6 months or (3) abdominal MRI including chemical shift imaging showed signal drop in opposed-phase or (4) the lesion showed an absolute washout > 60% in multiphasic CT. Lesions were diagnosed as metastases when (1) size of the lesion changed within 6 months or (2) abnormal ^18^F FDG uptake was shown at PET/CT in a known malignancy.

### Imaging protocol

All PCD-CT scans were performed on a dual-source photon-counting detector CT (NAEOTOM Alpha, Siemens Healthineers) as routine clinical acquisitions using a monophasic contrast injection protocol in portal venous phase. Each patient received a contrast bolus of 100 ml (Ultravist 300 mgI/mL, Bayer) that was injected via an antecubital vein (flow rate 4.0 ml/s) and followed by a saline bolus of 30 ml. Images were acquired in portal venous contrast phase (after fixed delay of 75 s after contrast injection).

All patients were scanned craniocaudally in a supine position during a single breath-hold. The following parameters were applied: acquisition mode with readout of spectral information (Abdomen QuantumPlus, Siemens Healthineers), 120-kV tube voltage, 0.5 s or 0.25 s, rotation time, 144 × 0.4 mm collimation.

A part of the patient cohort also received true non contrast CT, either within the same protocol prior to contrast application or within a separate acquisition, e.g., during FDG-PET or radiation planning.

### Image reconstruction

CTs were reconstructed from image raw data using a dedicated research software (ReconCT 16.0, Siemens Healthineers). For each patient, two VNC series, one conventional (VNC_conv_) and one pureCalcium (VNC_PC_) at a virtual monoenergetic level of 70 keV, iodine maps, and virtual monoenergetic image (VMI) series from 40 to 90 keV in 10-keV increments were reconstructed. The slice thickness and increment were 1.0 and 0.5 mm. A soft tissue kernel optimized for quantitative evaluation, Qr40, was used with an iteration strength of 3. Image reconstruction of TNC images was performed using SyngoVia (Syngo.via VB60A, Siemens Healthineers) and same slice thickness and increment as in the PCD-CT.

### Image analysis

**Fig. 1:**
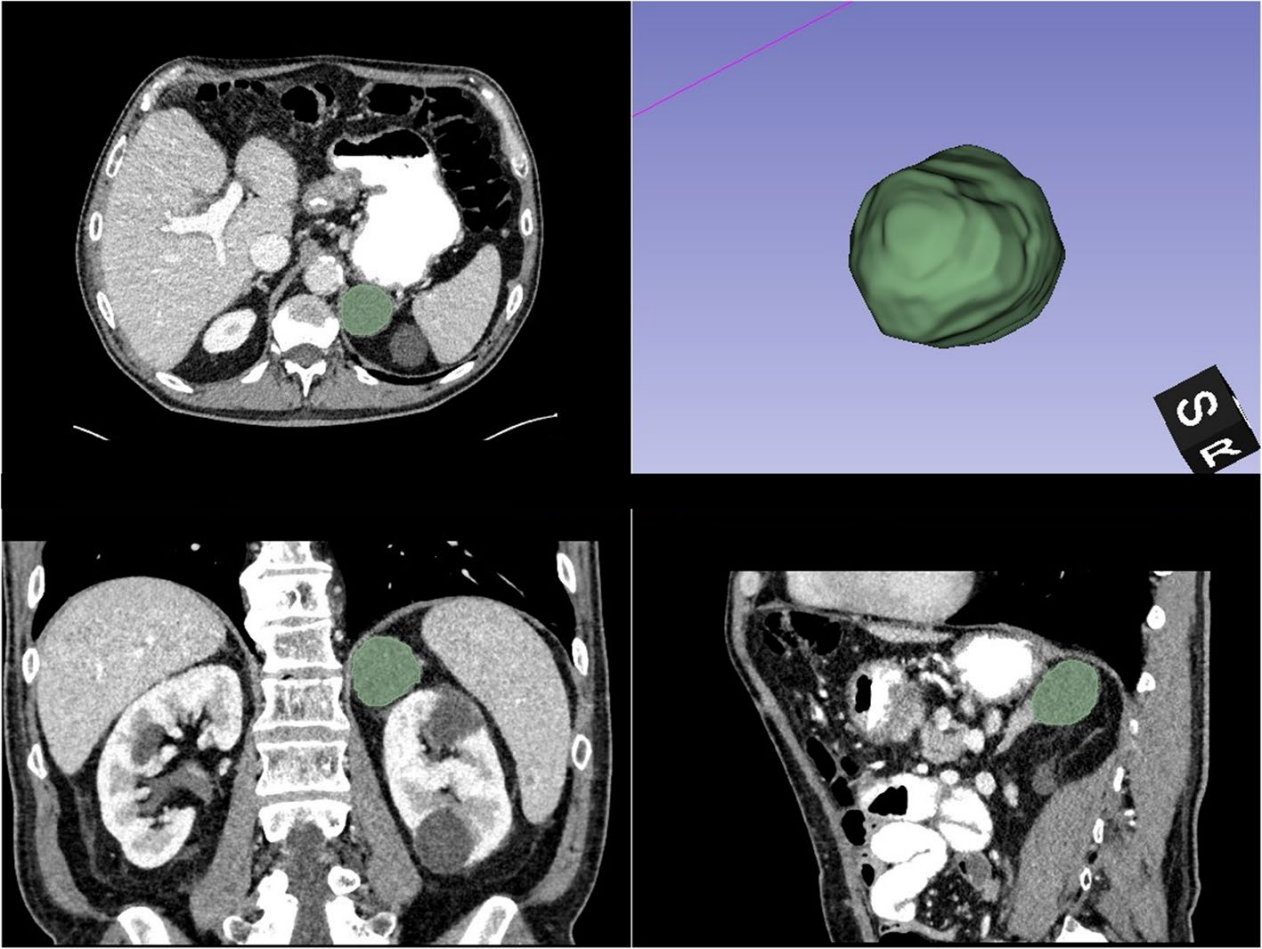
Example of semiautomatic segmentation of a left adrenal adenoma using 3D Slicer

Semiautomatic segmentation of adrenal lesions was performed by a board-certified radiologist (S.B.) with 7 years of experience in abdominal CT imaging using open-source software 3D Slicer (http://www.slicer.org [15]) (Fig. 1). Quantitative data were extracted after lesion segmentation for all reconstructions (VMI 40–90 keV, VNC_Conv_, VNC_PC_, and iodine maps) using pyradiomics (version 3.1.0 [16]). First-order features (mean HU values) were used for further analyses. True non-contrast (TNC) images were available for 49/90 patients (for 14/45 metastasis and 35/45 adenomas).

### Statistical analysis

Analysis of descriptive data and statistical analyses were performed using R (R Statistics, version 4.3.1, R Core Team, [17]), RStudio (version 2023.06.2 [18]), and Python 3.7.1 (www.python.org). Shapiro–Wilk tests were performed to check for normal distribution. Non-normally distributed data are presented as median and interquartile range (IQR), normally distributed data as mean (± standard deviation). Mann–Whitney *U* tests (non-normally distributed data) or *t*-tests (normally distributed data) were performed to compare between two groups. Bonferroni correction was performed for multiple testing. Bland–Altman plots were used to describe the similarity between VNC and TNC. Logistic regression models with imaging features (HU values from spectral data) as independent variables and outcome (adenoma vs. metastasis) as dependent variable were performed after splitting the cohort into training and test cohort (80/20). Five models were randomly built on the training cohort (fivefold cross-validation). After 5 models were trained and validated, the method gave as final trained model the one that obtained the best results. This model was then applied on the test cohort. ROC analyses were performed in R using the *pROC-package*. Data are shown for all established models (folds 1–5) as well as for the selected best model (test set). Area under the curve (AUC) is shown for the model that was applied on the test cohort. The optimal cutoff value was determined using the *cutpointr-package* in R. For feature selection, the Boruta package was applied in R. The Boruta algorithm is a wrapper method for feature selection and often applied in radiomics analyses [19]. The algorithm uses a random forest (RF)–based classification model to select the most important features. Statistically significant differences were assumed at *p*-values ≤ 0.05.

## Results

### Patient population

From the local database, 110 patients with adrenal lesions were identified. We excluded patients due to missing ground truth (*n* = 10), lesions too small to measure (*n* = 4), missing contrast-enhanced scan (*n* = 1), diagnosis of adrenal carcinoma (*n* = 1), and missing raw data (*n* = 4).

Finally, 90 patients (36 female assigned at birth, mean age 67.8 years [range 39–87]) were included in this study. Twenty-six patients had bilateral adrenal lesions; in these cases, we analyzed the left side (except for one case with calcification in the left adrenals). Baseline characteristics are shown in Table 1.

**Table 1:** Baseline characteristics

	Metastasis (*n* = 45)	Adenoma (*n* = 45)	*p* value
*n*, female (%)	20 (44.4)	16 (35.6)	0.395
Age, years (mean [range])	67.8 (39–87)	67.8 (45–87)	1.000
Side (measured), left (%)	35 (77.8)	37 (82.2)	0.603
BMI, kg/m^2^ (median [IQR])	24.18 (21.19–29.67)	27.49 (24.87–30.51)	0.051
CTDI_Vol_, mGy·cm, median [IQR])	7.37 (6.06–10.06)	8.67 (6.27–10.90)	0.418
Cancer origin	Lung cancer: *n* = 28 Renal / bladder cancer: *n* = 5 Colorectal cancer: *n* = 2 Breast cancer: *n* = 2 Lymphoma: *n* = 2 Ductal adenocarcinoma of the pancreas: *n* = 2 Esophagastric Junction Cancers (AEG): *n* = 2 Cancer of unknown primary (CUP): *n* = 1 Endometrial cancer: *n* = 1	n.a	n.a
Lipid-rich vs. lipid-poor	n.a	Lipid-rich: *n* = 35 (77.8%)	n.a
Mean lesion volume (mm^3^)	9757.53 (3493.1–11,510.3)	4626.2 (1140.4–6317.7)	0.001

*BMI* body mass index, *CTDI* computed tomography dose index, *IQR* interquartile range

### Quantitative analysis

**Fig. 2:**
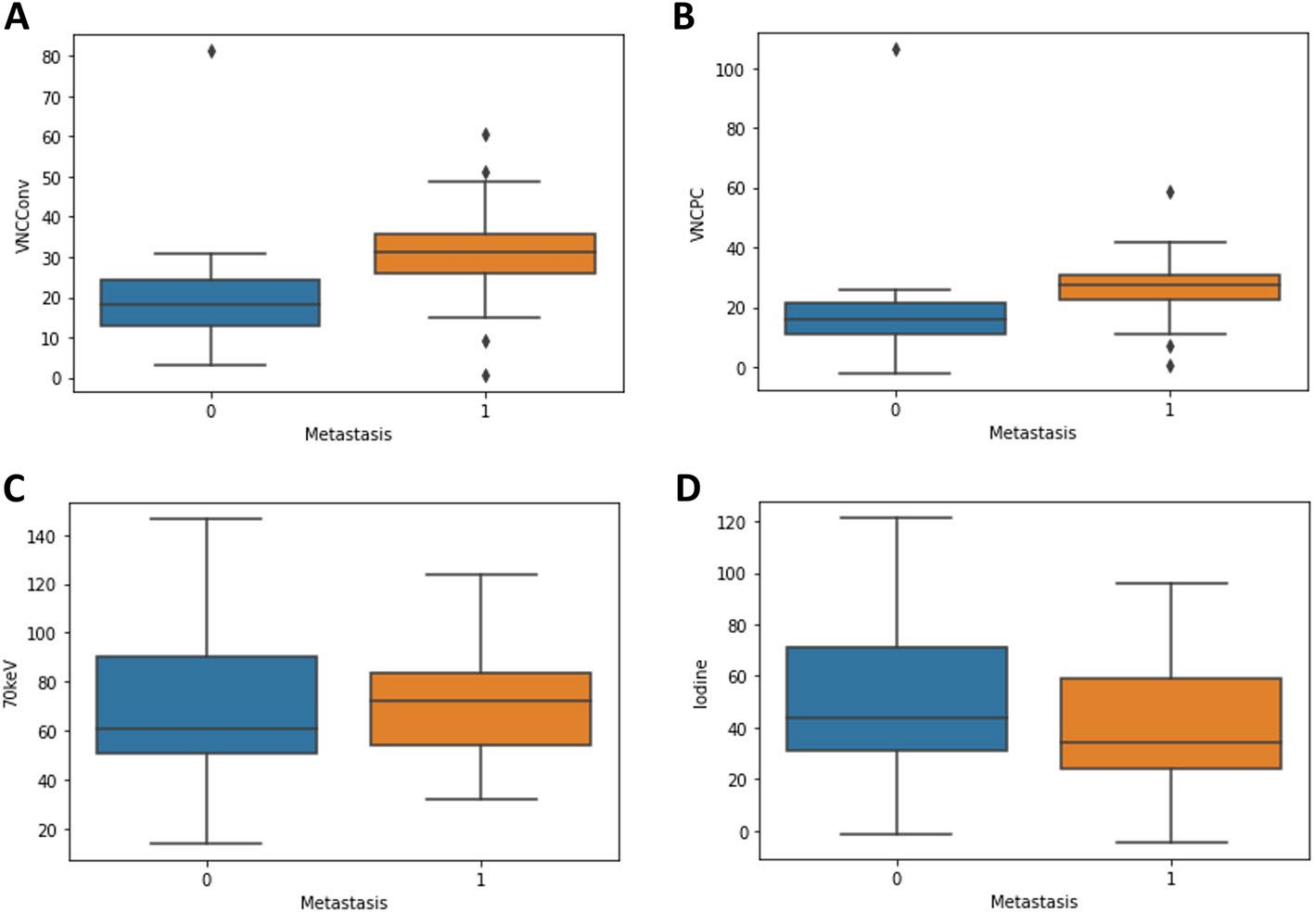
Boxplots for quantitative analyses of mean CT values **a** VNC_Conv_ (*p* = 0.007), **b** VNC_PC_ (*p* = 0.007), **c** 70 keV (*p* = 1.000) and **d** iodine density maps (*p* = 0.726) in adrenal lesions and differentiation between metastases and adenomas. 0 = no metastasis/adenoma; 1 = metastasis; VNC_Conv_ = conventional virtual non-contrast; VNC_PC_ = Virtual non-contrast pure calcium

Adrenal adenomas showed significantly lower median CT values in VNC_Conv_ reconstructions (18.15 [12.92–24.26] vs. 31.28 [26.00–35.89], *p* = 0.007) and in VNC_PC_ reconstructions (16.00 [11.02–21.56] vs. 27.22 [22.59–30.64], *p* = 0.007) compared to adrenal metastases (Fig. 2, Table 2).

**Table 2:** Mean CT values at different reconstructions in metastasis and adenoma

	Metastasis	Adenoma	*p* value
40 keV	149.53 (106.61–218.87)	161.57 (120.27–245.01)	1.000
50 keV	110.42 (81.03–151.41)	111.60 (88.78–172.80)	1.000
60 keV	86.70 (67.02–109.59)	78.93 (65.59–121.20)	1.000
70 keV	72.09 (53.89–83.88)	60.78 (50.77–90.37)	1.000
80 keV	61.11 (46.43–69.44)	49.82 (41–14–69.98)	1.000
90 keV	54.06 (42.88–60.03)	42.88 (34.88–58.47)	0.296
Iodine maps	33.93 (23.93–58.80)	43.92 (30.74–71.31)	0.726
VNC_Conv_	31.28 (26.00–35.89)	18.15 (12.92–24.26)	*0.007*
VNC_PC_	27.22 (22.59–30.64)	16.00 (11.02–21.56)	*0.007*
TNC	19.12 (7.52–22.77)	4.28 (− 3.24–7.11)	*0.021*

Data shown as median (interquartile range); *p* value shown after Bonferroni correction

*VNC* virtual non-contrast, *VNC*_*Conv*_ conventional VNC algorithm, *VNC*_*PC*_ pure calcium VNC algorithm, *TNC* true non-contrast

**Fig. 3:**
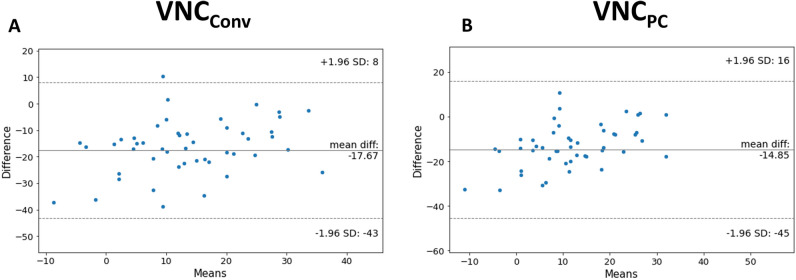
Bland Altman plots for difference between TNC and different VNC algorithms ­(**a** VNC_Conv_ and ­**b** VNC_PC_) in the subgroup of patients with available TNC (*n* = 49). TNC = true non-contrast; VNC_Conv_ = conventional virtual non-contrast; VNC_PC_ = Virtual non-contrast pure calcium

For patients with available TNC images (*n* = 49; metastasis = 14, adenoma = 35), Bland Altman plots showed a mean difference of 14.85 for VNC_PC_ reconstructions and of 17.67 for VNC_Conv_ reconstructions with higher values for both VNC algorithms compared to TNC (Fig. 3).

### Feature selection

**Fig. 4:**
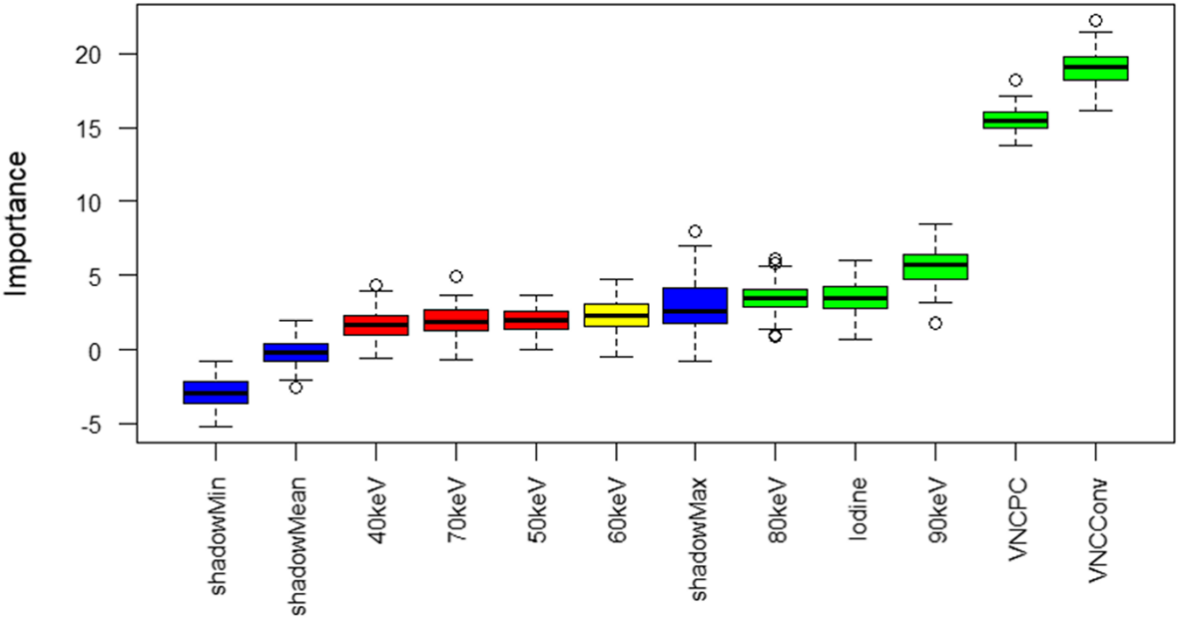
Feature importance in a Random Forest Model (Boruta)

Using the Boruta random forest method for feature selection, VNC_Conv_ and VNC_PC_ were selected as the features with the highest importance (Table 3, Fig. 4). Also, iodine maps as well as VMI 80 and 90 keV were important features in this model yet with lower importance.

**Table 3:** Feature importance

	Mean importance	Median importance	Minimum importance	Maximum importance	Decision
40 keV	1.688556	1.688494	− 0.57961552	4.320842	Rejected
50 keV	1.950700	1.926037	0.02061232	3.640417	Rejected
60 keV	2.358565	2.223441	− 0.53112638	4.766701	Tentative
70 keV	1.911188	1.849627	− 0.70735873	4.901965	Rejected
80 keV	3.405728	3.447088	0.90215446	6.125980	Confirmed
90 keV	5.585501	5.702772	1.76194257	8.457138	Confirmed
Iodine maps	3.491482	3.478601	0.70263658	5.992867	Confirmed
VNC_Conv_	19.038321	19.040796	16.09838496	22.228905	Confirmed
VNC_PC_	15.508909	15.486995	13.79535125	18.198172	Confirmed

*VNC* virtual non-contrast, *VNC*_*Conv*_ conventional VNC algorithm, *VNC*_*PC*_ pure calcium VNC algorithm

### Logistic regression models

**Fig. 5:**
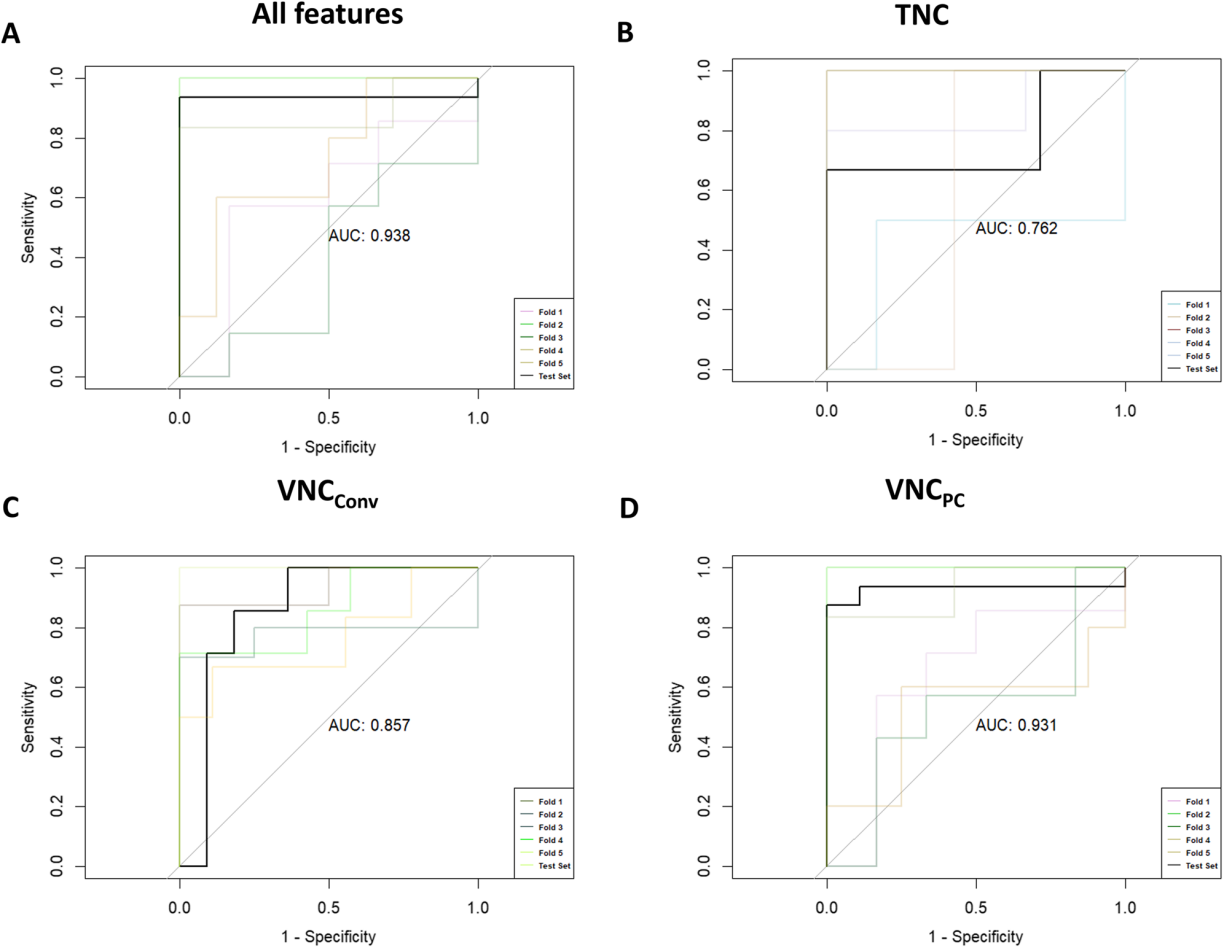
ROC analysis using metastasis as dependent variable and either **a** all features, **b** TNC, **­c** VNC_Conv_, or ­**d** VNC_PC_ as independent variables. Data are shown for all five trained models (folds 1–5) and for the best model that was selected after validation and then applied to the test cohort (test set). AUC is shown for the model that was applied to the test cohort. TNC = true non-contrast; VNC_Conv_ = conventional virtual non-contrast; VNC_PC_ = Virutal non-contrast pure calcium

Logistic regression models showed that use of all available spectral data had the best ability to discriminate between adrenal adenomas and adrenal metastases (AUC = 0.938) (Fig. 5). Used independently, TNC showed an AUC of 0.762, VNC_Conv_ reconstructions showed an AUC of 0.857, and VNC_PC_ reconstructions an AUC of 0.931 in ROC analyses (Fig. 5). Table 4 gives an overview of different cutoff values for VNC_Conv_ and VNC_PC_ reconstructions and TNC and their sensitivity and specificity as well as accuracy and precision for discrimination between adenomas and metastases. Using for example 26 HU in the VNC_Conv_ reconstruction as a cutoff value, there is a sensitivity of 86.7% and a specificity of 75.6% for the correct diagnosis. For TNC, an optimal cutoff value of 10.8 was observed in our cohort and showed best discrimination between benign and malign adrenal lesions with a sensitivity of 71.4% and a specificity of 82.9%.

**Table 4:** Best cutoff values

Threshold	Specificity	Sensitivity	Accuracy	Precision
VNC_PC_				
21.8	80.0	77.8	78.9	79.6
22.5	82.2	75.6	78.9	80.9
22.9	84.4	73.3	78.9	82.5
23.4	86.7	71.1	78.9	84.2
24.4	91.1	66.7	78.9	88.2
VNC_Conv_				
25.2	82.2	80.0	81.1	81.8
25.8	84.4	77.8	81.1	83.3
26.0	86.7	75.6	81.1	85.0
27.3	88.9	73.3	81.1	86.8
28.5	93.3	68.9	81.1	91.2
29.4	95.6	66.7	81.1	93.8
TNC				
10.8	82.9	71.4	79.6	62.5

*VNC* virtual non-contrast, *VNC*_*PC*_ pure calcium VNC algorithm, *VNC*_*Conv*_ conventional VNC

algorithm, *TNC* true non-contrast

## Discussion

Although the mean differences to TNC are smaller on VNC_PC_ than on VNC_Conv_ series, there is still an overestimation of CT values in the assessment of adrenal lesions. Nevertheless, the use of VNC facilitates the discrimination of adenomas and metastases on contrast-enhanced scans, using a higher threshold for discrimination (e.g., 26 HU instead of the established 10 HU for TNC).

The diagnostic workup of incidental adrenal lesions on contrast-enhanced CT scans is a well-known challenge in radiology. In most cases, further imaging (e.g., adrenal CT or MRI) or follow-up is recommended. This leads to increased costs due to additional testing, which can also lead to increased radiation exposure and/or delayed diagnosis. In the era of DECT and PCD-CT, one might think that virtual non-contrast imaging could solve this problem. However, similar to previous studies on DECT, the first published experiences on PCD-CT also reported that VNC over- or underestimates CT values compared to TNC [1, 9, 20].

Therefore, VNC has not been recommended for routine clinical use because even a discreet discrepancy in CT values may have a significant impact on the diagnosis and further workup of adrenal lesions [4, 11]. Similar to previous studies, we also reported significant overestimation of VNC_Conv_ compared to TNC with a mean difference of about 17 HU.

In this study, we aimed also to assess the value of a new and promising VNC algorithm (VNC_PC_) in the diagnosis of adrenal lesions [13]. Whereas the discrepancy in CT values compared to TNC is smaller using this new algorithm, there is still a mean difference of about 15 HU. A mean deviation of 15 HU might be too high in the assessment of adrenal adenomas and might entail incorrect diagnoses.

Therefore, it is necessary to address this problem in the near future and to improve algorithms of VNC. However, VNC reconstructions might still have value in clinical routine currently if a “new” (different) threshold for VNC reconstructions is agreed on. Logistic regression analyses in this work revealed higher optimal thresholds for VNC in the discrimination between adenomas and metastases. For example, using a threshold of 26 HU instead of the established 10 HU for TNC results in high sensitivity and specificity for differentiating between adenomas and metastases. In our study, specificity and sensitivity were even higher using, e.g., 26 HU in VNC reconstructions compared to 10 HU in TNC. Our analyses reported different “optimal” cutoff values for VNC_Conv_ and VNC_PC_, which are presented in Table 4. They differ regarding specificity and sensitivity. With lower HU values, sensitivity increases while specificity decreases and vice versa. In our opinion, 26 HU was the best “compromise” between specificity and sensitivity for VNC_Conv_. Therefore, we decided to choose this value. However, further studies with larger patient cohorts must confirm the results of this study and define the “final” optimal cutoff value. An integration of this threshold in clinical routine also requires further analyses; the findings of well-established 10 HU as the best threshold in TNC in our cohort support the validity of our examinations. Yet, one major challenge might be the standardization of the results in VNC reconstructions. Due to rapid technical developments, different VNC algorithms exist. Therefore, standardization of VNC pipelines or alternatively a harmonization of data will be crucial aspects for upcoming studies with larger patient cohorts. Especially multi-center studies with consented protocols are necessary to confirm the results of this study and to redefine the threshold for the diagnosis of adrenal lesions in contrast-enhanced CT.

The present study also analyzed the value of different imaging features derived from spectral imaging (e.g., iodine maps, VMI) in discriminating between adrenal adenomas and metastases. A previous study on DECT showed promising results for the combination of iodine density and VNC [1]. Other studies have also highlighted the discriminative power of fat fraction and radiomics [7, 8].

To our knowledge, no previous studies have addressed the value of spectral imaging for the discrimination between adrenal adenomas and adrenal metastases. Our study shows that adrenal adenomas and metastases have significantly different imaging characteristics (e.g., adenomas have lower CT values in VNC_Conv_ and VNC_PC_ compared to metastases), which is similar to previously reported results on DECT [1].

Logistic regression analysis and random forest classifiers identified VNC (both VNC_Conv_ and VNC_PC_) as the most important parameters in discriminating between adrenal adenomas and metastases.

In view of these results, it is very important to address the technical optimization of VNC algorithms to approximate CT values of TNC and/or the definition of a “new” (different) threshold for VNC. The possibility to safely replace TNC with VNC could save many CT or MRI scans in the workup of adrenal lesions, might accelerate the diagnosis, and potentially reduce psychological burden for the patients due to delayed clarification.

In contrast to the previous study on DECT [1] and also to the previous study on PCD-CT [11], we performed a complete 3D segmentation of the entire adrenal lesion and did not use only ROI-based measurements. We aimed to improve the diagnostic accuracy and avoid bias that may be introduced by ROI-based measurements. However, similar results with an over- and underestimation of CT values in VNC (compared to TNC) were also observed with this method. The spectral information used in this study is based on PCD-CT differentiating photon energy (above a certain level to eliminate electronic noise) in high and low, e.g., using one threshold to create two so-called bins. In the future, up to four bins (differentiation with three thresholds) will most likely be available according to the manufacturer. This could provide even more information, potentially leading to improved CT values in VNC reconstructions.

Body mass index (BMI) is known to affect image noise and image quality [21]. A previous study on PCD-CT showed that contrast-to-noise ratio (CNR) for detection of liver metastases did not differ in a wide range of BMI suggesting a preservation of CNR on a PCD-CT [22]. Similar results were also shown in a previous study analyzing the conspicuity of pancreatic ductal adenocarcinoma on a PCD-CT; this study also found no significant differences in CNR in portal venous phase for patients with higher and lower BMI [23]. The present study showed no significant differences in BMI between patients with adenomas and metastases; however, a tendency towards a lower BMI in the metastases cohort was observed. According to the results of the previous studies and the suggested CNR-preserving potential of PCD-CT in a wide range of BMI, we do not expect these findings to significantly influence the results of the present study (also due to the small patient cohort).

This study has limitations: first, this was a single-center study with inclusion of both—patients with known or suspected malignancies and multiphasic CT scans for assessment of adrenal lesions [24]. Second, TNC was only available for a subgroup of patients with a higher proportion of patients with adenomas which might introduce a bias. Third, the diagnosis of adenomas or metastases was not confirmed histopathologically, but by either further imaging (e.g., adrenal CT, unenhanced CT, MRI) or follow-up.

## Conclusion

VNC reconstructions tend to overestimate CT values in comparison to TNC in the assessment of adrenal lesions. However, there is still a high diagnostic accuracy for both, the conventional and the new calcium-preserving VNC algorithm in the discrimination of adrenal lesions, especially when elevating the established threshold of 10 HU to about 26 HU. Therefore, a “new” threshold for VNC reconstructions may safely discriminate adrenal lesions and might save further workup of unclear cases. Further studies with larger patient cohorts and multi-center studies using standardized methodology and data harmonization are necessary to confirm the results of this study.

## Abbreviations

DECT: Dual-energy CT
keV: Kiloelectronvolt
PCD-CT: Photon-counting detector CT
TNC: True non-contrast
VMI: Virtual monoenergetic images
VNC_Conv_: Conventional virtual non-contrast
VNC_PC_: Virtual non-contrast pure calcium

## Declarations

### Guarantor

The scientific guarantor of this publication is Josua Decker.

### Statistics and biometry

Several authors have significant statistical expertise.

### Study subjects or cohorts overlap

The study subjects or cohorts have not been previously reported.

### Methodology

• retrospective

• diagnostic or prognostic study

• performed at one institution
